# Assessing interventions promoting the uptake of cancer‐related genomic services within the Latino community: A scoping review using the RE‐AIM framework

**DOI:** 10.1002/cam4.7440

**Published:** 2024-07-11

**Authors:** Dayanna Ramirez Leon, Denise Martinez, Jessica Rivera Rivera, Lindsay Fuzzell, Susan Vadaparampil, Hannah Rogers, Sheryl Gabram, Cindy Snyder, Yue Guan

**Affiliations:** ^1^ Department of Behavioral, Social, and Health Education Sciences Rollins School of Public Health, Emory University Atlanta Georgia USA; ^2^ Department of Health Outcomes and Behavior H. Lee Moffitt Cancer Center and Research Institute Tampa Florida USA; ^3^ Woodruff Health Sciences Center Library Emory University Atlanta Georgia USA; ^4^ Georgia Center for Oncology Research and Education Atlanta Georgia USA

**Keywords:** cancer, evaluation, genetic counseling, genetic testing, Latino health, RE‐AIM

## Abstract

Cancer genomic services (CGS) can support genetic risk‐stratified cancer prevention and treatment. Racial/ethnic minority groups are less likely to access and utilize CGS compared with non‐Hispanic Whites. Little research has described characteristics of interventions targeted at CGS among Latinos. This scoping review aimed to (1) describe interventions promoting uptake of CGS among Latinos in the United States and Latin America, (2) describe intervention adaptations for Latino participants, and (3) summarize intervention implementation factors suggested by reach, effectiveness, adoption, implementation, and maintenance (RE‐AIM) framework. We conducted a search in English and Spanish of literature published between 2005 and 2022 across PubMed and Latin American and Caribbean Health Sciences Literature databases. Sixteen of 2344 papers met the inclusion criteria of the analysis. Efforts to promote CGS among Latino communities were limited in the US and lower in Latin America. This review highlights the need for in‐depth exploration of acculturation‐informed interventions and better reporting on implementation factors to enhance their scalability across diverse settings.

## Graphical abstract

Little research has described characteristics of interventions targeted at cancer genomic services (CGS) among Latinos. This scoping review aimed to (1) describe interventions promoting uptake of CGS among Latinos in the United States and Latin America, (2) describe intervention adaptations for Latino participants, and (3) summarize intervention implementation factors suggested by reach, effectiveness, adoption, implementation, and maintenance (RE‐AIM) framework. We conducted a search in English and Spanish of literature published between 2005 and 2022 across PubMed and Latin American and Caribbean Health Sciences Literature databases.

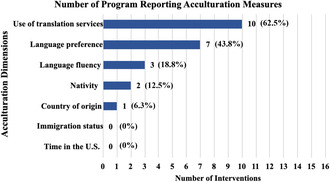

## 1 INTRODUCTION

The Latino population in the United States (US) is diverse, consisting of members with ancestry from multiple countries.^1^ Since 1970, the Latino population has grown more than sixfold and now makes up 19% (62.5 million individuals) of the national population.^2^ Cancer is the leading cause of death among this group, accounting for 20% of deaths.^3^ Earlier studies report that to non‐Hispanic Whites and Latinas have a higher incidence of triple‐negative breast cancer and are diagnosed at younger ages, factors that are associated with a higher likelihood of carrying hereditary genetic variants that increase cancer risk.^4^, ^5^, ^6^, ^7^ However, present research indicates that Latinas have a lower incidence and mortality rate of triple‐negative breast cancer compared with non‐Hispanic White women, suggesting that there are research gaps in knowledge surrounding hereditary cancer‐related incidence, mortality, and screening practices among Latinas.^8^, ^9^, ^10^ Despite this, the uptake of family history screening, genetic counseling, and genetic testing among Latina women remains low, even in specialty settings.^11^, ^12^, ^13^ As the Latino population continues to increase in the US, disparities in hereditary cancer‐associated cases and premature deaths will continue to widen.^2^

Cancer genomic services (CGS) can support risk management and reduce hereditary cancer‐related mortality.^14^, ^15^ These services have notable benefits particularly relevant to the Latino population. For example, brief family history assessments endorsed by national and public health organizations (e.g., United States Preventative Services Task Force [USPSTF] and Centers for Disease Control and Prevention [CDC]) now enable population‐based screening to identify families at high risk for *BRCA*‐associated cancers and Lynch syndrome (LS).^16^, ^17^, ^18^, ^19^, ^20^ The National Comprehensive Cancer Network (NCCN) recommends genetic testing for patients diagnosed with colorectal cancer under the age of 50 and those with a personal history of tumor testing or a family history suggesting Lynch Syndrome.^21^ Implementing cancer genetic screening is critical as individuals who carry a *BRCA1/2* mutation have significantly increased lifetime risks for breast (50%–80%) and ovarian cancer (10%–40%), and individuals with LS have an increased lifetime risk of developing colorectal cancer (22%–74%), endometrial cancer (15%–71%), and ovarian cancer (4%–20%).^22^, ^23^, ^24^ Potentially lifesaving prevention and treatment options are available to mutation carriers; thus, early detection is key to improvement of cancer outcomes (e.g., risk‐reducing surgeries, conventional chemotherapy, enhance screening).^5^, ^23^, ^24^ However, Spanish‐preferring Latinas are half as likely as Whites to have discussed genetic counseling (GC) or genetic testing (GT) with a health provider. English‐preferring Latinas and Spanish‐preferring Latinas have greater odds of having unmet need for discussing GT with providers (OR = 2.44 and OR = 7.28, respectively).^25^ Similarly, findings from a national health survey found that among those who reported completing GT, only 23% of Latinos reported completing GT for cancer screening compared 42% of non‐Hispanic White respondents.^26^ The Hispanic Community Health Study, a longitudinal cohort study, found that only 3.3% of participants reported ever completing GT.^27^ These findings demonstrate the underutilization of CGS among the Latino community. Efforts to expand CGS beyond urban cancer specialty settings that serve predominantly non‐Hispanic White populations have been exceedingly slow, which will likely lead to further worsening of health disparities.^28^, ^29^, ^30^, ^31^

Various factors may contribute to the low uptake of CGS, such as cost, inadequate insurance coverage, lack of awareness on the part of the patient and/or provider, and limited availability of screening services.^32^ Additionally, language barriers and immigration status may pose unique challenges for Latinos in accessing these screening services. While many Spanish‐speaking patients prefer language‐concordant GC, only 6% of US genetic counselors provide services in Spanish, and 60% use a bilingual medical interpreter.^33^, ^34^, ^35^ Spanish‐preferring patients who receive GC in English or through interpreters have reported low hereditary breast and ovarian cancer knowledge, uncertainty about the purpose of testing, feeling overwhelmed, and inaccurate understanding of cancer risk and risk management options.^36^, ^37^ An analysis of the 2005 Health Information Trends Survey also found that individuals with immigrant status were less likely to report a family history of cancer, possibly resulting from fewer opportunities to learn about their family history due to separation from extended or immediate family members.^38^ Providers may also forgo referring high‐risk Latinos to cancer‐related GC due to concerns about access, language, and cultural barriers, thus reducing opportunities for increasing awareness and knowledge about cancer risk and genetic screening among Latinos.^39^ These barriers are partially associated with the community's level of acculturation—the degree to which the majority culture is adopted by a minority culture.^40^ However, it is unclear to what extent existing strategies considered acculturation levels or made adaptations for the Latino community. While there is ample research that explores the individual‐level predictors (i.e., income, education level, etc.) for CGS attitudes and knowledge among minority groups,^41^, ^42^, ^43^ it is unknown how many existing interventions have aimed to promote the uptake of CGS among Latinos. Understanding the characteristics of successful interventions geared toward Latino communities can facilitate the implementation of future interventions in broader settings and address persistent racial and ethnic inequities in CGS implementation, utilization, and access.^44^

RE‐AIM (reach, efficacy/effectiveness, adoption, implementation, maintenance) is an implementation framework designed to guide the planning and evaluation of programs to support program implementation in realistic settings.^45^ Application of the RE‐AIM framework could facilitate systematic evaluation of intervention strategies implemented, with a special focus on implementation strategy adaptations and components that support intervention sustainability and scalability.^46^ This scoping review aimed to (1) describe interventions to promote uptake of cancer‐related genomic services among Latino populations in the US and Latin America, (2) describe the extent to which these interventions have adapted to increase their fit for Latino community needs, and (3) summarize the components suggested by RE‐AIM implementation framework that are associated with potential scalability of these strategies.

## 2 METHODS

### 2.1 Study design

**FIGURE 1:**
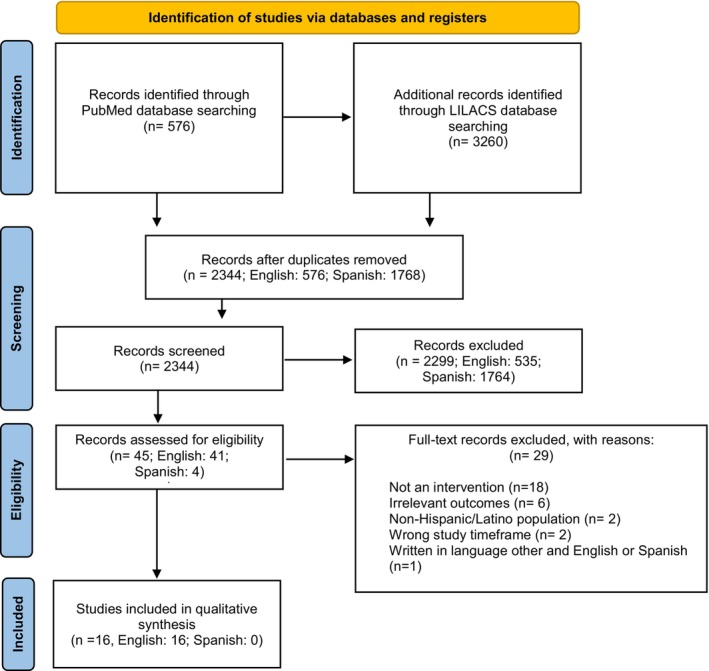
PRISMA flow diagram.

A scoping review, informed by Arksey and O'Malley's scoping study framework, was performed as the research team anticipated a limited number of primary interventions reporting CGS interventions among Latino communities indicated by current racial/ethnic disparities in genetic databases.^47^ A scoping review was conducted to map the existing body of evidence on interventions aimed to address this health disparity to highlight new ways of understanding it.^47^, ^48^ This paper utilizes the preferred reporting items for systematic reviews and meta‐analyses (PRISMA) guidelines for reporting on scoping reviews.^49^ The PRISMA strategy is outlined in Figure 1. The current scoping review did not involve human subjects and therefore was exempt from the IRB approval process.

### 2.2 Inclusion/exclusion criteria

The present analysis included interventional designs to promote the uptake of CGS that occurred within the US and/or Latin America after September 2005 to capture the clinical interventions that occurred after the USPSTF recommendation that women whose family history is associated with an increased risk for deleterious mutations in *BRCA1* or *BRCA2* genes be referred for GC and evaluation for BRCA testing. Papers were included if they described evidence‐based interventions aimed at increasing the utilization of cancer genomic services, were conducted in the U.S. or Latin America, and were human studies that included participants that are identified or self‐identified as Latino/Hispanic.

For the purposes of this study, cancer genomic services are defined as clinical services that include hereditary risk assessment of cancer, cancer genetic education or counseling, and cancer GT.^50^ We defined ‘evidence‐based’ interventions as intervention strategies and methods informed by or derived from peer‐reviewed documented evidence (i.e., data‐based, research‐based, or scientifically‐based). Adaptations were defined as intentional modifications to achieve a better fit between an intervention and a new context, such as tailoring language, visuals, or literacy level.^51^, ^52^ We defined ‘intervention’ as a strategy or program to promote the uptake of cancer‐related genomic services. In this scoping review, Latino was defined as having origins or descent from Argentina, Bolivia, Brazil, Chile, Colombia, Costa Rica, Cuba, Dominican Republic, Ecuador, El Salvador, Guatemala, Honduras, Mexico, Nicaragua, Panama, Paraguay, Peru, Puerto Rico, Uruguay, or Venezuela.^1^ While additional gender‐neutral terms, such as Latinx and Latine may also describe this community, Latino is often used as a gender‐neutral option and is widely accepted by the community.^53^ For the purposes of this study, acculturation measures included language fluency, language preference, immigration status, nativity, country of origin, and time/number of generations living in the US.^54^ Lastly, the intervention levels assessed were patient, provider, and organization (none of which were mutually exclusive).

Papers were excluded from the analysis if they were not written in English or Spanish, were not peer‐reviewed journal articles (including conference and meeting abstracts, commentaries, editorials, literature reviews, scoping reviews, and systematic reviews), performed tumor testing without direct patient involvement, conducted genetic screening/testing with the sole purpose of determining eligibility for a clinical trial, or did not provide full‐text access.

## 3 SEARCH STRATEGY AND STUDY SELECTION

Our search strategy (Appendix 1: Tables A1 and A2) was developed with the assistance of the library services of the Woodruff Health Sciences Center Library at Emory University. The PubMed search terms included free‐text and MESH terms for Latino (and terms that captured multiple nationalities and identities within the Latinx community), genetic screening, cancer, and cancer screening on PubMed.

Our search terms were then adapted for the Latin American and Caribbean Health Sciences Literature (LILACS) database by translating the English search terms to Spanish. Due to the limitations in the search function in the LILACS database, researchers chose the broadest search terms to produce results relevant to the scoping review (cáncer y genética/o [cancer and genetics] and tumor y genética/o [tumor and genetics]). The scoping review included interventions published in English or Spanish published within September 2005 to September 2022.

## 4 DATA EXTRACTION AND STUDY SELECTION

Titles and abstracts were double‐screened for inclusion by two of five reviewers (D.R., Y.G., D.M., J.R.R., or L.F.). Remaining studies were single‐screened at full‐text review by one of three reviewers (D.R., Y.G., or D.M.). Discrepancies were resolved through discussion.

The data extraction form was developed by the study team (D.R., Y.G.) and uploaded into the Covidence systematic review system. The research study characteristics extracted included authors, year of publication, country, study interventions to promote uptake of cancer‐related genomic services, intervention characteristics, study population, number of participants, and the main findings. The outcomes of interest included the uptake of genetic risk assessment (individuals who completed genetic risk screening/individuals who could have been screened), uptake of genetic education/counseling (individuals who completed GC services/individuals eligible for GC services), and uptake of GT (individuals who completed GT/individuals eligible for GT).^55^ The acculturation measures identified in the previous section were also extracted. D.R., Y.G., and D.M. extracted qualitative and quantitative data based on the presence (1) or absence (0) of components for each RE‐AIM dimension (Table 3). Operationalization of each RE‐AIM dimension was informed by examples listed on the RE‐AIM website (https://re‐aim.org/learn/what‐is‐re‐aim/) and previous applications of the framework in health‐related systematic reviews.^55^, ^56^, ^57^ Frequency counts and percentages were recorded for each RE‐AIM component, and means were calculated for each RE‐AIM indicator using Microsoft Excel 365.

The study team (D.R., D.M., Y.G.) pilot tested the data extraction form with three studies and reached consensus among coders for each article. The lead author (DR) independently coded the rest of the eligible studies. The study team (D.R., D.M., Y.G.) met regularly to resolve any coding discrepancies and operationalize the RE‐AIM framework.

## 5 QUALITY ASSESSMENT

Rigor was assessed using the Effective Public Health Practice Project's Quality Assessment Tool for Quantitative Studies, chosen due to the tool's validity and reliability in assessing observational and experimental interventions.^58^, ^59^ Criteria assessed for study quality include study design, data collection methods, withdrawals, intervention integrity, and analyses.

## 6 RESULTS

### 6.1 Study characteristics

Of the 576 search results produced in the PubMed parent scoping review, only 16 met the inclusion criteria. Of the 3260 search results produced in LILACS, 1768 papers remained after duplicates were removed, and 0 met the inclusion criteria. Table 1 summarizes the descriptive characteristics of the included interventions (*n* = 16). Most interventions took place within the US (*n* = 13),^12^, ^60^, ^61^, ^62^, ^63^, ^64^, ^65^, ^66^, ^67^, ^68^, ^69^, ^70^, ^71^ and the remaining interventions were conducted in Latin American countries, including Brazil (*n* = 2)^72^, ^73^ and Mexico (*n* = 1).^74^ Of the 16 interventions included, 14 (87.5%) of the interventions were evidence‐based^12^, ^60^, ^62^, ^63^, ^64^, ^65^, ^66^, ^67^, ^68^, ^69^, ^71^, ^72^, ^73^, ^74^ and 2 (12.5%) interventions were not evidence‐based.^61^, ^70^ The most frequently hereditary cancer syndromes were hereditary breast and ovarian cancer (*n* = 14, 77.8%),^12^, ^60^, ^61^, ^63^, ^64^, ^65^, ^66^, ^67^, ^69^, ^70^, ^71^, ^72^, ^73^, ^74^ Lynch syndrome (*n* = 6, 37.5%)^61^, ^64^, ^68^, ^70^, ^72^, ^73^ and melanoma (*n* = 2, 12.5%).^62^, ^64^ Various studies reported a study sample that was predominately highly educated (*n* = 6, 37.5%),^60^, ^62^, ^63^, ^64^, ^67^, ^70^ had limited knowledge regarding genetic risk and testing (*n* = 4, 25.0%),^61^, ^63^, ^71^, ^74^ had completed mammogram screening (*n* = 3, 18.8%),^12^, ^60^, ^67^ had a personal (*n* = 3, 18.8%)^12^, ^71^, ^74^ or family history (*n* = 2, 12.5%)^65^, ^72^ of cancer, or lacked health insurance coverage (*n* = 2, 12.5%).^28^, ^68^ Five (31.3%) were cross‐sectional interventions,^12^, ^60^, ^61^, ^72^, ^73^ 4 (25.0%) interventions were randomized controlled trials,^62^, ^63^, ^66^, ^71^ 3 (18.8%) were cohort studies,^65^, ^68^, ^69^ 2 (12.5%) were non‐randomized experimental interventions,^67^, ^70^ 1 was a case–control study,^64^ and 1 (6.3%) was a mixed‐methods study.^74^ A detailed summary of each intervention and its characteristics can be found in Table 1.

**TABLE 1:** Summary and characteristics of included studies.

Study	Design description	Screened hereditary cancer syndromes	RE‐AIM dimensions					Quality assessment
			Reach	Effectiveness	Adoption	Implementation	Maintenance	
Abul‐Husn et al., 2021, USA^61^	Cross‐sectional study No control group	HBOC, LS, familial hypercholesterolemia (FH), Hereditary transthyretin amyloidosis (hATTR)	*N* = 74 Male: 13 (17.4%); Female: 61 (82.4%); Mean age: 58 years Age range: 28–83 years Latino: 31%	GRA: N/A GC: 95% GT: 100% Other outcomes: Evaluated characteristics of participants who opted to receive their results.	Program was adopted by genetic counselors and Spanish translators at a university medical center.	Adaptations: patient‐level; Spanish translators were available for patients and language preference was measured. Piloted the return of results to patients prior to complete implementation of the program. Barriers: outdated patient contact information and patients leaving the health system.	N/A	Weak
Anderson et al., 2015, USA^60^	Cross‐sectional study No control group	HBOC	*N* = 243 Age range: 25–69 years Female: 100% Latino: 26.2%	GRA: 100% Latino GRA: 100% GC: N/A GT: N/A Other outcomes: Evaluated characteristics of women who did not want to discuss individualized risk results	Delivered by 2 research staff members in 2 federally qualified health centers.	Adaptations: provider‐level; Breast Cancer Risk Screening tool was created using the Gail model, the Claus model, and pedigree assessment tool to evaluate FH for HBOC risk. Training: Research staff received a 4‐hour tool‐specific training.	N/A	Weak
Blazer et al., 2021, Mexico^74^	Mixed methods study No control group	HBOC	*N* = 1321 Male: 17 (1%); Female: 1304 (99%) Latino: 100% Varying healthcare coverages (private, no coverage, and national program coverage)	GRA: 100% GC: N/A GT: 100% Other outcomes: Evaluated implementation of low‐cost BRCA testing program and genomic cancer risk provider knowledge	Delivered in four clinic settings (1 university, 2 cancer centers, 1 hospital) by 11 staff members (6 geneticists, 2 oncologists, 1 gynecologist, 1 general physician, 1 genetic laboratory clinician).	Adaptations: patient‐ and provider‐levels; Spanish translations for consent and protocol documents, roundtable discussions conducted in Spanish. Training: genomic cancer risk, case report forum, patient cancer risk questionnaire Piloted program in one location before expanding to the other three. Cost: $20 BRCA testing per case Facilitators: clinics engaging in site visits from the research team. Barriers: English language educational materials for clinicians with Spanish‐language preference, health care finance, adequately trained workforce, and population‐based registries in Mexico, cost for supplies, sample storage, support staff, and shipping	All physicians who received training continued to conduct GCRA. One site trained additional clinicians for more sustained services. Some clinicians left their institutions to implement GCRA services at other institutions.	Weak
Campacci et al., 2017, Brazil^72^	Cross‐sectional study Risk survey was administered in person, over the telephone, or via letter	HBOC, LS	*N* = 20,000 Age range: 18–79 years Average age: 51 ± 9.45 years Female: 100% Latino: 100%	GRA: 100% GC: N/A GT: N/A Other outcomes: Create and validate the primary screening questionnaire (PSQ) to identify people with risk for hereditary cancer predisposition syndrome	Delivered by nurses with experience in cancer genetics in 12 mobile units across 381 cities and in a cancer hospital.	Adaptations: patient‐level; educational materials were written in simple language for comprehension and materials were provided in Portuguese.	N/A	Weak
Conley et al., 2021, USA and Puerto Rico^71^	Randomized controlled trial Intervention: culturally targeted educational booklet about GC/GT Control: BC survivorship fact sheet	HBOC	*N* = 52 Average age: 54 ± 9 years Female: 100% Latino: 100% Born in Puerto Rico: 56% Born in Columbia: 21% Born in Cuba: 17%	GRA: 78.8% GC ○ Intervention: 65% ○ Control: 15% GT ○ Intervention: 94% ○ Control: 100% Other outcomes: Pre/post HBOC knowledge and emotional distress	Delivered by researchers and genetic counselors at a cancer center in the USA and a university research center in PR.	Adaptations: patient‐level; educational booklet was adapted from a brief English brochure about GC/GT with additional content regarding GC (definition, process, and benefits) and GT (process, timeframe, and financial aid resources); booklet was translated into Spanish. Barriers: low provider referral and patient awareness, limited access, and language and cultural barriers to genetic services. Facilitators: scheduling GT and follow‐up	N/A	Moderate
Hay et al., 2018, USA^62^	Randomized controlled trial Intervention: MC1R test offer Control: usual care	MC1R (melanoma risk)	*N* = 499 Age range: 19–85 years Mean age: 54 years Male: 103 (21%); Female: 376 (79%) Latino: 48.5%	GRA: N/A GC: 46.49% Latino GC: 38.4% GT: 33.5% Latino GT = 23.1% Other outcomes: Prevalence and interest in MC1R testing in diverse primary care population	Delivered by bilingual project assistants at a university medical center.	Adaptations made at the patient‐level; recruitment materials were translated into Spanish; printed version of the website content was provided to patients without internet access. Barriers include lack of knowledge, lower genomic literacy, and lack of confidence in the medical system.	N/A	Moderate
Kukafka et al., 2022, USA^63^	Randomized controlled trial Intervention: decision aid for patients and clinicians Control: patient education	HBOC	*N* = 187 Age range: 21–75 years Female: 100% Latino: 46.6% Can consent in English or Spanish	GRA ○ Intervention: 99.0% ○ Control: 100% GC ○ Intervention: 19.8% ○ Control: 11.6% GT ○ Intervention: 36.6% ○ Control: 24.4%	Delivered at an academic medical center by research team and included 67 clinicians.	Adaptation: patient‐level; consent and patient education materials translated into Spanish. Decision aid and risk navigation tool were pilot tested among ethnically diverse women at high‐risk for BC.	N/A	Moderate
Lee et al., 2005, USA^64^	Case–control study	HBOC, LS, melanoma	*N* = 7316 Female: 100% Non‐English speaking: 25% Latino: 26%	GRA: N/A GC: 26% GT: 27% Other outcomes: Demographic characteristics of participants and effective patient recruitment methods	Delivered by a genetic counselor, a part‐time physician, and a clerk at a public health hospital.	Adaptations: patient‐ and organization‐levels; FH questionnaire was translated into Russian, Chinese and Spanish; survey was revised to include additional questions about other cancers in family, Ashkenazi Jewish ancestry, and permission for direct contact if patient did not have a PCP. Training: in‐service education on GC and *BRCA* testing for hereditary cancers Barriers: provider education, patient recruitment, assembly of a multidisciplinary team, and adapting genetic education to a population with marginal health literacy.	N/A	Weak
McGuinness et al., 2019, USA^12^	Cross‐sectional study No control group	HBOC	*N* = 3055 Age range: 29–91 years Mean age: 58 years Female: 100% Latino: 76.7%	GRA: 85.9% Latino GRA: 90.3% GC: N/A GT: 4.6%	Delivered by research team at an academic medical center.	Adaptation: patient‐level; Created 10‐question assessment using Gail model to assess eligibility for HBOC GT. Inclusion criteria specified participants could speak Spanish. No mention of translating services used. Barriers: limitations of current FH guidelines in identifying women who would benefit from GC/GT services for HBOC.	N/A	Weak
Mette et al., 2016, USA^70^	Non‐randomized experimental study Video teleconferencing vs. in person GC	HBOC, LS	*N* = 353 Age ≥ 50 years: 58% Latino: 56.6% English‐preferring: 84%	GRA: 100% GC: 100% GT: 79.8% Other outcomes: Compare effectiveness of telemedicine and in person delivery models on genetic risk assessment and GC education.	Delivered in four clinic settings by healthcare providers, genetic counselors, and oncologists.	Adaptation: patient‐level; genetic risk training was provided to healthcare providers; survey instrument was translated into Spanish and adapted to be accessible to patients with low literacy levels; language preference was measured, and the intervention was conducted in English and Spanish. Training: staff from 4 clinics received education on genetic services Patient satisfaction surveys were used to assess implementation. Facilitators: GC videoconferencing to Texas‐Mexico border Barriers: cost of services, limited accessibility of medical history due to language barriers, and under use of health services.	N/A	Weak
Nogueira et al., 2021, Brazil^73^	Cross‐sectional study No control group	HBOC, LS	*N* = 675 Latino: 100%	GRA: N/A GC: N/A GT: 91%	Delivered by a psychologist, lab technician, administrative staff, oncogeneticist, and general geneticist in a clinic setting.	No adaptations made. Language used for intervention not specified. Training: clinicians were trained on FH screening and hereditary cancer referral. Costs: administrative analysts created a cost center to monitor resource use and prepare financial reports. Barriers: making the service financially sustainable and overcoming testing taboos.	Discussed 3 potential strategies to improve sustainability of the program.	Weak
Pasick et al., 2016, USA^66^	Randomized controlled trial Intervention: phone call Control group: educational brochure	HBOC	*N* = 88 Age range: 28–69 years Female: 100% Latino: 36.3% Foreign‐born: 25.0%	GRA: 100% GC ○ Intervention: 68.2% ○ Control: 47.7% GT: 9.8%	Delivered by telephone information specialists. Individuals were referred to genetic counselors (GC).	Adaptation: patient‐ and organizational levels; Pedigree Assessment Tool to administer over the phone and mirrored EWC intake questions to fit capabilities of information specialist team (high school level education); offered GC in Spanish. Facilitators: having high school‐level education specialists screen callers and having a GC assistant conduct a follow‐up.	Mention of collaboration with Cancer Risk Program to screen women but status of funding for GC referrals after the study is unclear.	Moderate
Schonberg et al., 2020, USA^67^	Non‐randomized experimental study Pre and post groups	HBOC	*N* = 337 Age range: 40–49 years Female: 100% Mean age: 44.1 ± 2.9 Latino: 9.2%	GRA: 19% GC: 6% GT: N/A Other outcomes: Examine the effect of providing women in their 40s and their PCPs with a 2‐page personalized BC risk report before a PC visit	Delivered by Primary Care Clinician (PCC) at a university PCP.	No adaptations made. Program delivery occurred at the patient‐level. Training: conversation aids required minimal training	N/A	Moderate
Snedden et al., 2020, USA^68^	Cohort study 3 sequential cohorts of newly diagnosed patients with CRC (PRE, PERI, POST)	LS	*N* = 381 PRE (*N*) = 272 PERI (*N*) = 25 POST(*N*) = 84 Male: 230 (60.4%), Female: 151 (39.6%) Latino: 62.5%	GRA ○ PRE: 30.5% ○ PERI: 64.0% ○ POST: 58.3% GC ○ PRE: 46.7% ○ PERI: 75.0% ○ POST: 80.0% GT ○ PRE: 46.7% ○ PERI: 75.0% ○ POST: 80.0%	Delivered by gastroenterologists, medical oncologists, surgeons, and pathologists in a clinical setting. The pathologists were responsible for verbally requesting and confirming the order for testing.	Adaptations: organizational and provider‐level; implementation of the Universal Tumor Screening (UTS) algorithm. UTS designed to reflect IHC screening for patients. Training: printed and verbal education among all responsible providers Barriers: pathologists not adhering to implemented algorithm with complete compliance.	N/A	Weak
Soewito et al., 2022, USA^65^	Retrospective cohort study GCs provided educational sessions to health providers and community groups near the TX‐MEX border. No control group	HBOC	*N* = 1595 Age range: 18+ years Male: 224 (14%), Female: 1371 (86%) Latino: 80% Low SES, literacy, and educational attainment levels	GRA: N/A GC: 86% GT: 86%	Program delivered by Community Health Workers at the home clinic, locally on periodic outreach trips (occurring in 8‐week intervals), or through video‐teleconferencing.	Adaptations: patient‐level; Language preference was measured, and intervention was conducted in English and Spanish by CHWs; GC was conducted by phone when video‐teleconference sites were restricted. Program demonstrated equivalent patient satisfaction and outcomes in video‐teleconference vs. in person counseling. Barriers: challenges with VUS communication. Facilitators: video‐teleconferencing and telephone delivery during COVID‐19 pandemic.	N/A	Weak
Walker et al., 2021, USA^69^	Historical control study Intervention: Genetic Testing Station (GTS) Control: traditional referral method	HBOC, pancreatic ductal adenocarcinoma (PDAC)	*N* = 223 Mean age: 64.6 ± 12 years Male: 116 (52%), Female: 107 (48%) Latino: 5% Spanish‐preferring: 7 (3%)	GRA: 100% GC: 66% GT: 92%	Intervention occurred at 1 oncology clinic. Program delivered by Nurse Navigators (NN), New Patient Coordinators (NPC), Genetic Counseling Assistants (GCA), and Genetic Counselors (GC).	Adaptation: patient‐ and organizational level. Implemented GTS referral system. GT was supported by philanthropic funds at no cost to the patient. Barriers: required GC availability for patient referrals. Facilitators: introducing the concept of germline testing in advance of the initial clinic appointment to facilitate appointment scheduling.	N/A	Weak

Abbreviations: FH, family history; GC, genetic counseling; GRA, genetic risk assessment; GT, genetic testing; HBOC, hereditary breast and ovarian cancer; LS, lynch syndrome; PCP, primary care provider.

### 6.2 Characteristics of the intervention

Most interventions occurred at the patient‐level (*n* = 12, 75.0%),^12^, ^61^, ^62^, ^63^, ^64^, ^65^, ^66^, ^69^, ^70^, ^71^, ^72^, ^74^ followed by the organizational level (*n* = 6, 37.5%),^60^, ^64^, ^66^, ^68^, ^69^, ^74^ and the provider‐level (*n* = 4, 25.0%).^60^, ^65^, ^68^, ^74^ Seven interventions addressed multiple levels.^60^, ^64^, ^65^, ^66^, ^68^, ^69^, ^74^ Of these interventions, 13 (81.25%) piloted the use of cancer risk assessment tools (electronic, printed, or administered via phone call),^12^, ^60^, ^61^, ^64^, ^65^, ^66^, ^67^, ^68^, ^69^, ^70^, ^71^, ^72^, ^74^ 12 (75.0%) provided educational materials on GC/GT, cancer, and genetic risk among providers and/or patients (outreach events, brochures, and in‐service trainings),^60^, ^62^, ^63^, ^64^, ^65^, ^66^, ^68^, ^69^, ^70^, ^71^, ^72^, ^74^ and 3 (18.75%) provided printed individualized cancer risk results to patients.^60^, ^61^, ^67^

### 6.3 Strategies to address acculturation

**FIGURE 2:**
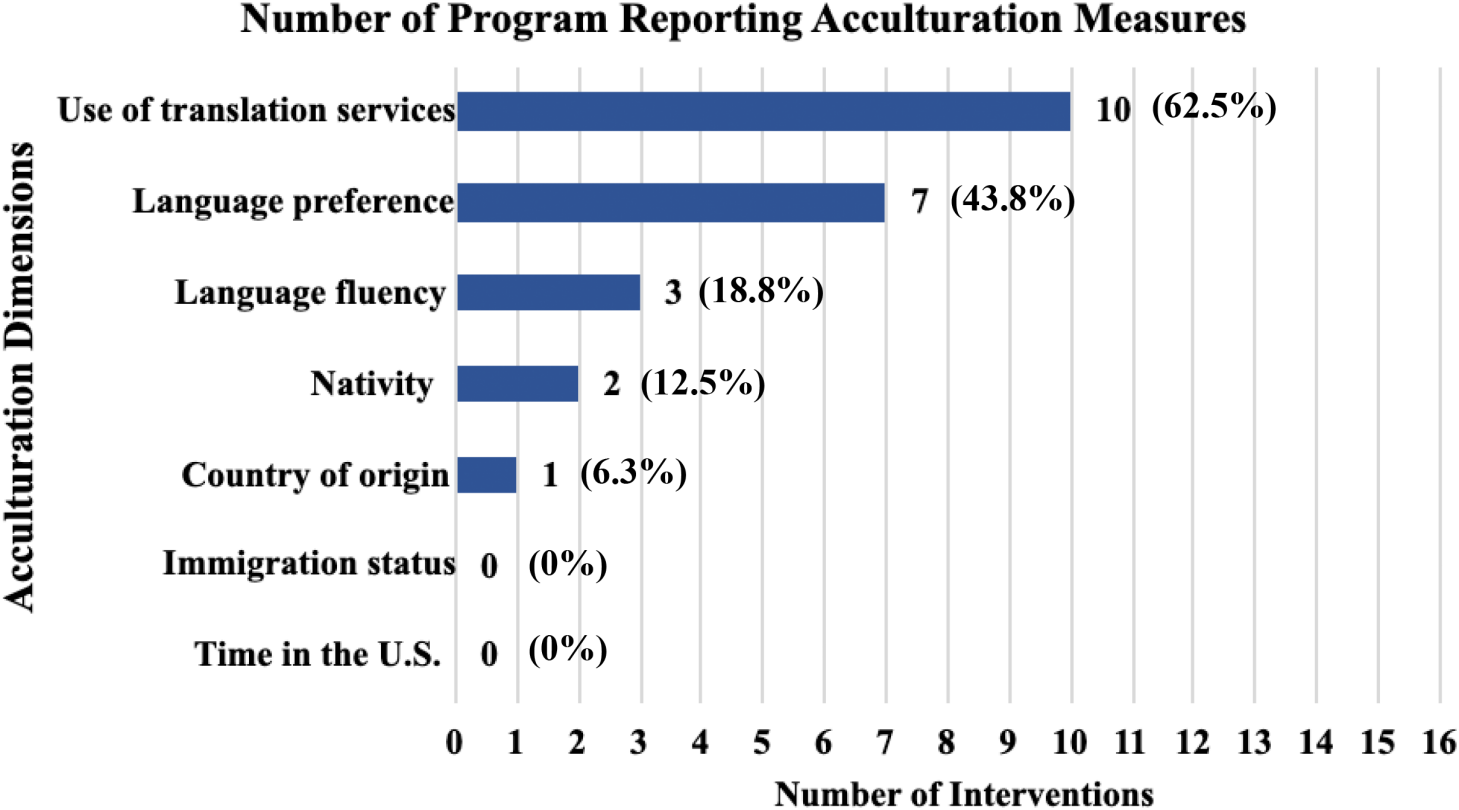
Number of interventions reporting acculturation measures (*n* = 16).

Most interventions considered acculturation issues to some extent during their design and implementation phases (*n* = 11, 68.8%).^61^, ^62^, ^63^, ^64^, ^65^, ^66^, ^69^, ^70^, ^71^, ^73^, ^74^ As seen in Figure 2, common strategies to address acculturation include use of translation services (*n* = 10, 62.5%),^61^, ^62^, ^63^, ^64^, ^65^, ^66^, ^70^, ^71^, ^73^, ^74^ measuring language preference (*n* = 7, 43.8%)^61^, ^63^, ^65^, ^69^, ^70^, ^71^, ^74^ or fluency (*n* = 3, 18.8%),^62^, ^66^, ^74^ and documenting nativity (*n* = 2, 12.5%)^62^, ^71^ and country of origin (*n* = 1, 6.3%).^74^

### 6.4 RE‐AIM dimensions

Table 3 summarizes the number and percentage of interventions reporting each RE‐AIM dimension and component. The most reported dimensions of the implementation framework were reach (69.8%) and efficacy (70.9%). The least reported dimension of the framework was maintenance (10.4%).

### 6.5 Reach

Baseline sample sizes ranged from 52 to 20,000 (median = 359) participants. All interventions reported the proportion of Latino/a participants (range = 5%–100%). Most interventions reported characteristics such as sex (*n* = 15, 93.8%)^12^, ^60^, ^61^, ^62^, ^63^, ^64^, ^65^, ^66^, ^67^, ^68^, ^69^, ^71^, ^72^, ^74^ and age range (*n* = 10, 62.5%).^12^, ^60^, ^61^, ^62^, ^63^, ^65^, ^66^, ^67^, ^69^, ^72^ Some reported characteristics such as language literacy and language preference. Eight (50.0%) interventions only targeted female patients and screened for hereditary breast and ovarian cancer syndrome.^12^, ^60^, ^63^, ^64^, ^66^, ^67^, ^71^, ^72^

All interventions reported their methods for identifying the target population for the intervention and sample size (*n* = 16, 100%), and most interventions outlined their inclusion criteria (*n* = 15, 93.8%)^12^, ^60^, ^61^, ^62^, ^63^, ^64^, ^65^, ^66^, ^67^, ^68^, ^69^, ^70^, ^71^, ^72^, ^74^ and participation rate (*n* = 10, 62.5%).^12^, ^60^, ^61^, ^62^, ^63^, ^66^, ^67^, ^69^, ^70^, ^74^ Patients were recruited through an electronic health record^61^, ^68^, ^73^ or registry,^68^, ^71^ recruiting in clinic settings^12^, ^60^, ^62^, ^63^, ^64^, ^65^, ^69^, ^71^, ^72^, ^74^ or outreach events,^71^ by patient referral,^70^, ^73^ by mail,^67^ or through a call center.^66^ Few interventions, however, outlined their exclusion criteria (*n* = 4, 25.0%)^62^, ^67^, ^68^, ^69^ or the characteristics of individuals who refused to participate (*n* = 6, 37.5%).^60^, ^62^, ^63^, ^66^, ^68^, ^69^ The reach dimension had an overall reporting proportion of 69.8%.

### 6.6 Efficacy/effectiveness

Of the 16 interventions included, the uptake rate of genetic risk assessment screening was available for 10 (62.5%) interventions (uptake rate range: 19%–100%).^12^, ^60^, ^63^, ^66^, ^67^, ^68^, ^69^, ^70^, ^71^, ^72^ Three additional studies completed genetic risk screening but did not provide information on how many participants could have been screened.^64^, ^73^, ^74^

Eleven interventions (68.8%) reported the uptake rate of GC/education (range: 6%–95%).^61^, ^62^, ^63^, ^64^, ^65^, ^66^, ^67^, ^68^, ^69^, ^70^, ^71^ Four additional interventions reported how many patients were eligible for GC/education; however, they did not report how many participants completed GC.^60^, ^72^, ^73^, ^74^

Thirteen interventions (81.3%) reported the uptake rate of GT (uptake rate range: 4.6%–100%).^12^, ^61^, ^62^, ^63^, ^64^, ^65^, ^66^, ^68^, ^69^, ^70^, ^71^, ^73^, ^74^ Three interventions did not measure GT uptake as an intervention outcome.^60^, ^67^, ^72^ Overall, 70.9% of the efficacy/effectiveness domain was reported by the interventions included in the review. Assessing the uptake of CGS among Latinos is challenging as only five studies reported uptake of genetic risk assessment among Latinos.^12^, ^60^, ^71^, ^72^, ^74^ Two studies reported uptake of GC among Latinos,^62^, ^71^ and four reported uptake of GT among Latinos.^62^, ^71^, ^73^, ^74^

### 6.7 Adoption

All interventions (*n* = 16, 100.0%) provided a description of the intervention location. Most interventions (*n* = 11, 68.8%) occurred in one site location^12^, ^60^, ^61^, ^62^, ^63^, ^64^, ^65^, ^67^, ^69^, ^70^, ^72^ and 5 (31.3%) occurred across multiple site locations.^66^, ^68^, ^71^, ^73^, ^74^ Intervention settings included universities (*n* = 12, 75.0%),^12^, ^61^, ^62^, ^63^, ^65^, ^66^, ^67^, ^68^, ^69^, ^70^, ^71^, ^74^ oncology hospitals (*n* = 3, 18.8%),^72^, ^73^, ^74^ cancer centers (*n* = 2, 12.5%),^71^, ^74^ primary care clinics (*n* = 2, 12.5%),^60^, ^73^ a national research center (*n* = 1, 6.3%),^74^ a general hospital (*n* = 1, 6.3%),^64^ and a state department of health (*n* = 1, 6.3%).^66^ Fourteen interventions (87.5%) described the staff who delivered the intervention and their level of expertise.^60^, ^61^, ^62^, ^64^, ^65^, ^66^, ^67^, ^68^, ^69^, ^70^, ^71^, ^72^, ^73^, ^74^ Delivery agents include PCPs, GCs, nurses, community health workers, and research assistants with multiple years of experience. Of these 14 interventions, only 1 reported their method to identify the intervention delivery agent.^74^ Three interventions (18.8%) reported the cost characteristics of the intervention implementation, such as the materials needed to complete GT, the cost of genetic tests, and printed educational materials.^71^, ^73^, ^74^ None of the interventions included in the review reported the adoption rate of the intervention. Overall, the interventions included reported 48.0% of the components in the adoption dimension.

### 6.8 Implementation

All interventions (*n* = 16, 100%) reported the intervention type and intensity of the intervention. Seven interventions (43.8%) conducted training for the personnel delivering the intervention^60^, ^63^, ^67^, ^68^, ^70^, ^72^, ^73^ and 2 (12.5%) interventions piloted health education materials prior to the start of the intervention.^71^, ^74^ Eleven interventions (68.8%) reported barriers to implementation,^12^, ^61^, ^62^, ^64^, ^65^, ^68^, ^69^, ^70^, ^71^, ^73^, ^74^ the most common being lack of adequately trained workforce, financial barriers to GC/GT, lack of GT/GC knowledge among patients, limited family history knowledge, and language barriers. Only 6 (37.5%) interventions reported facilitators to implementation,^65^, ^66^, ^69^, ^70^, ^71^, ^74^ including reducing health literacy required to comprehend GT/GC educational materials and scheduling GT/GC appointments earlier in the intervention process. Most interventions (*n* = 7, 43.8%) delivered the intervention within the current workflow of the organization.^12^, ^64^, ^66^, ^68^, ^69^, ^70^, ^72^ Six interventions (37.5%) reported the extent to which the protocol was delivered as intended^62^, ^66^, ^67^, ^68^, ^69^, ^71^ and 1 (6.3%) intervention reported the cost of implementation.^74^ Most interventions (*n* = 14, 87.5%) made adaptations to the intervention.^12^, ^60^, ^61^, ^62^, ^63^, ^64^, ^65^, ^66^, ^68^, ^69^, ^70^, ^71^, ^72^, ^74^ Of these interventions, adaptions in 7(43.8%) addressed only one level of implementation,^12^, ^61^, ^62^, ^63^, ^70^, ^71^, ^72^ 6 (37.5%) addressed two levels,^60^, ^64^, ^65^, ^66^, ^68^, ^69^ and 1 (6.3%) addressed three levels (levels assessed: patient, provider, organization).^74^ Most adaptations made were related to various measures of acculturation. Other adaptations included designing intervention materials that mirror current workflow of intervention setting (*n* = 4, 25.0%),^60^, ^66^, ^69^, ^74^ reducing required literacy of health education materials (*n* = 4, 25.0%),^66^, ^70^, ^71^, ^74^ incorporating changes to health education materials after piloting (*n* = 3, 18.8%),^64^, ^70^, ^71^ and providing no‐/low‐cost GC/GT (*n* = 2, 12.5%).^69^, ^71^ Overall, the interventions reported an average of 49.2% components of the implementation dimension.

### 6.9 Maintenance

Of the 16 interventions, only 3 (18.8%) assessed intervention outcomes 6 months after the implementation^63^, ^64^, ^67^, ^74^ and 2 (12.5%) interventions reported the current status of the intervention.^73^, ^74^ One intervention reported an expansion of the program to other site locations and among other clinical practitioners.^74^ Another intervention is exploring strategies to improve the sustainability of the program.^73^ No intervention reported the cost associated with the maintenance of the program. Overall, the interventions reported an average of 12.5% of the components of the maintenance dimension.

### 6.10 Quality assessment

Based on the quality assessment tool criteria, 11 interventions (68.7%) were of weak quality,^12^, ^60^, ^61^, ^64^, ^65^, ^68^, ^69^, ^70^, ^72^, ^73^, ^74^ 5 interventions (32.3%) were of moderate quality,^62^, ^63^, ^66^, ^67^, ^71^ and none of the included interventions were of strong quality (Table 2). The moderate‐quality interventions utilized a RCT and a pre‐/post‐cohort study design.

**TABLE 2:** Descriptive characteristics of interventions.

Studies (*n* = 16)	
Country	
USA	13 (81.3%)
Brazil	2 (12.5%)
Mexico	1 (6.3%)
Study design	
Cross‐sectional study	5 (31.3%)
Mixed methods	1 (6.3%)
Randomized controlled trial	4 (25.0%)
Case–control study	1 (6.3%)
Non‐randomized experimental study	2 (12.5%)
Cohort study	3 (18.8%)
Quality rating	
Strong	0
Moderate	5 (32.3%)
Weak	11 (68.7%)
Intervention setting	
Academic institution	12 (75.0%)
Cancer center	2 (12.5%)
Oncology hospital	3 (18.8%)
Primary care clinic	2 (12.5%)
National research center	1 (6.3%)
General hospital	1 (6.3%)
State department of health	1 (6.3%)
Intervention level	
Patient‐level	12 (75.0%)
Provider‐level	4 (25.0%)
Organization‐level	6 (37.5%)
Number of levels addressed in study	
1	7 (43.8%)
2	6 (37.5%)
3	1 (6.3%)
Evidence‐based design	
Yes	13 (81.3%)
No	2 (12.5%)

**TABLE 3:** Proportion of cancer‐related genetic service interventions among Latinos reporting RE‐AIM (*n* = 16 interventions).

RE‐AIM Dimensions and Components	Proportion reporting
Reach	
Method to identify target population	16 (100%)
Inclusion criteria	15 (93.8%)
Exclusion criteria	4 (25%)
Sample size	16 (100%)
Participation rate	10 (62.5%)
Characteristics of nonparticipants	6 (37.5%)
Average of overall reach dimension	69.8%
Efficacy/Effectiveness	
Uptake of genetic risk assessment	10 (62.5%)
Uptake of genetic counseling and education	11 (68.8%)
Uptake of genetic testing	13 (81.3%)
Average of overall efficacy/effectiveness dimension	70.9%
Adoption	
Description of intervention location	16 (100%)
Description of staff who delivered intervention	14 (87.5%)
Method to identify target delivery agent	1 (6.3%)
Level of expertise of delivery agent	14 (87.5%)
Adoption rate	0 (0%)
Characteristics of cost implementation	3 (18.6%)
Average of overall adoption rate	48.0%
Implementation	
Intervention type and intensity	16 (100%)
Extent protocol delivered as intended (%)	6 (37.5%)
Measures of cost of implementation	1 (6.3%)
Conducted training courses for intervention delivery agents	7 (43.8%)
Piloted health education materials	2 (12.5%)
Reported facilitators to implementation	6 (37.5%)
Reported barriers to implementation	11 (68.8%)
Adaptation made for intervention	14 (87.5%)
Average of overall implementation dimension	49.2%
Maintenance	
Assessed outcomes ≥6 months post‐intervention	4 (25%)
Current status of program	2 (12.5%)
Cost of maintenance	0 (0%)
Average of overall maintenance dimension	12.5%

## 7 DISCUSSION

Few published interventions promoting the uptake of GC/GT services among Latino populations in the U.S. have been reported, and fewer in Latin America. CGS are rare in many Latin American countries, possibly because genetic counseling is not a formally recognized discipline in these countries, limited CGS expertise among healthcare professions, few genetic counseling training programs, and lack of CGS services among socioeconomically disadvantaged populations.^73^, ^74^, ^75^, ^76^ Of 2344 publications, 16 interventions (0.68%) conducted among Latino communities were published. Of these interventions, none were uniquely identified through LILACS or published in Spanish, highlighting the lack of CGS uptake research in Latin America.

The most common intervention delivery method was incorporating an existing or modified family history‐based screening tool within the current clinic setting workflow. While most interventions addressed multiple levels, they primarily focused on the patient level (75%). Comparatively, a recent systematic review conducted across the U.S. population found that only 17 of 44 (39%) interventions designed to promote the uptake of CGS focused solely on the patient level.^77^ The results of this review may be inconsistent with interventions conducted among the overall US population due to the prominence of acculturation‐informed intervention design in our review. To increase the fit of the intervention for Latino patients, 11 interventions included acculturation measures and adaptations to address language barriers in clinic settings and demonstrated higher uptake of CGS. These findings suggest that patient‐level implementation and adaptation may be uniquely necessary for communities that experience language barriers in health care settings. Future interventions may explore the role of acculturation measures at provider/organizational levels such as assessing the impact of cultural humility trainings on healthcare providers' cross‐cultural interactions, examining the availability and quality of translation services within healthcare organizations to assess communication and comprehension gaps, and implementing strategies to engage patients from diverse backgrounds in CGS uptake.

Commonly applied acculturation‐informed strategies included use of translation services, measuring language preference and fluency, and documenting nativity and country of origin. However, important acculturation measures, such as immigration status and time/generations in the US, went unreported. These findings were consistent with prior literature in healthcare settings.^54^ While considering language barriers is necessary to address gaps in uptake among the Latino community, it may also be valuable to consider less‐reported factors. For example, time/generations in the US and immigration status may provide important context for providers regarding patient knowledge of GC/GT and incomplete patient family history.^38^ In this review, interventions^12^, ^67^, ^68^ that did not implement adaptations to address varying levels of acculturation generally reported lower rates of uptake of CGS compared with the interventions^61^, ^65^, ^66^, ^70^, ^71^, ^74^ that implemented acculturation‐informed adaptations. Studies that did not report any language‐based adaptations^60^, ^67^, ^69^ had much lower recruitment of Latinos in the study sample compared with interventions that did incorporate these adaptations. Inclusion of additional acculturation measures can inform the design of culturally relevant health education programs and interventions that account for the heterogeneity of the Latino population.

Adapting established screening methods to current patient intake processes facilitated implementation and promoted adoption of screening tools among staff, regardless of educational background or expertise. There is promising evidence that implementing existing screening tools into current organizational workflows can promote the delivery and uptake of CGS. However, most interventions lacked maintenance details of the intervention, making the status of the program unclear. Additionally, there was a lack of reporting of intervention implementation factors that were associated with program scalability and sustainability. Future interventions may benefit from the application of an implementation framework, such as RE‐AIM, to ensure factors of scalability and sustainability are measured and reported. The use of implementation frameworks in intervention design can further our ability to reproduce interventions outside of academic institutions and reduce access to care barriers, which is essential to address the paucity of interventions aimed at the Latino community despite the broad and inclusive search conducted for the present scoping review.

Regarding increasing access among Latino communities, the interventions showed varying success. Of the interventions included, 5 (31.25%) interventions had a sample that was 100% Latino, and 2 (12.5%) interventions included samples that were less than 25% Latino. Most interventions (*n* = 8, 50%) included exclusively female study populations. Interventions that addressed access barriers highlighted by Latino communities, such as cost, high participant burden, timeliness of care, and language barriers, showed high reach potential for CGS uptake. Existing research also highlights the importance of cultural factors, such as religion, in the formation of genetic fatalism beliefs.^78^ Although the research base is limited, current literature suggest that creating culturally curated intervention strategies can facilitate the uptake of CGS among Latino communities by addressing perceived concerns and negative emotional effects.^78^ Future research may focus on intentionally recruiting from Latino communities and measuring CGS uptake among men to improve intervention methods that benefit the community at large. Implementing the use of community‐based participatory research and transdisciplinary methods can further develop our knowledge base on addressing existing inequities in CGS by emphasizing mutual learning and minimizing bias.^79^, ^80^

Our review has some limitations. Our analysis included all relevant interventions that reported inclusion of Latino participants, regardless of the proportion. As such, the findings of some interventions may not be applicable to Latino populations if the community was underrepresented in the study population. Additionally, many studies did not report characteristics that highlight the diversity of Latino study participants, thus limiting our ability to analyze uptake rates across ethnicities, language preference groups, or immigration statuses. Research focused on understanding or addressing racial/ethnic disparities in CGS should consider reporting uptake by such characteristics to identify hidden trends in large racial/ethnic groups. Despite these limitations, our review included literature searches in both English‐ and Spanish‐language databases to characterize interventions designed to promote the uptake of CGS among Latino communities in the US and Latin America. Acculturation‐informed adaptations may improve the fit of future interventions with the Latino community and promote the uptake of CGS services. The lack of consensus on the role acculturation plays on the health experiences of Latinos highlights the need for further explore acculturation‐informed intervention design and implementation to better understand how to operationalize acculturation and analyze its impact on health outcomes. Usage of the RE‐AIM framework showed that various measures of implementation are underreported, thus limiting application in other settings and scalability. Designing future interventions using implementation frameworks may improve intervention scalability of successful strategies and increase utilization of CGS services among Latino communities.

## AUTHOR CONTRIBUTIONS

**Dayanna Ramirez Leon:** Conceptualization (lead); data curation (equal); formal analysis (lead); investigation (equal); methodology (equal); software (equal); visualization (lead); writing – original draft (lead); writing – review and editing (equal). **Denise Martinez:** Formal analysis (equal); validation (equal); writing – review and editing (equal). **Jessica Rivera Rivera:** Formal analysis (equal); validation (equal); writing – review and editing (equal). **Lindsay Fuzzell:** Formal analysis (equal); resources (equal); validation (equal); writing – review and editing (equal). **Susan Vadaparampil:** Methodology (supporting); resources (equal); writing – review and editing (equal). **Hannah Rogers:** Data curation (lead); resources (lead); software (equal); writing – review and editing (equal). **Sheryl Gabram:** Conceptualization (supporting); supervision (supporting); writing – review and editing (equal). **Cindy Snyder:** Conceptualization (supporting); supervision (supporting); writing – review and editing (equal). **Yue Guan:** Conceptualization (equal); formal analysis (equal); funding acquisition (lead); methodology (equal); project administration (equal); supervision (equal); validation (equal); writing – review and editing (equal).

## FUNDING INFORMATION

PI (Guan) received a research grant from the Emory University Rollins School of Public Health Dean's Pilot and Innovation 2022 Grant to pay authors (Dayanna Ramírez and Denise Martinez) for the time spent preparing the manuscript.

## CONFLICT OF INTEREST STATEMENT

No authors have conflict or interests to report.

## Data Availability

All data and materials are available upon request (Dayanna Ramirez, dayanna.ramirez@emory.edu).

## APPENDIX 1 SEARCH STRATEGY

**TABLE A1:** Search terms for PubMed.

Concept	Specific search terms
Latine	(hispanic*[tiab] OR “hispanic american*”[tiab] OR “hispano*”[tiab] OR “latine*”[tiab] OR “latina*”[tiab] OR “latin”[tiab] OR “latinu*”[tiab] OR “latino”[tiab] OR “latinx*”[tiab] OR “latin american*”[tiab] OR “latin america”[Mesh] OR “spanish speak*”[tiab] OR “mexico*”[tiab] OR “Mexico”[Mesh] OR “cuban*”[tiab] OR “peruvian*”[tiab] OR “dominican*”[tiab] OR “brazilian*”[tiab] OR “central american*”[tiab] OR “costa rican*”[tiab] OR “guatemalan*”[tiab] OR “honduran*”[tiab] OR “uruguayan*”[tiab] OR “argentina”[Mesh] OR “argentine”[tiab] OR “argentinian”[tiab] OR “argentinean”[tiab] OR “panamanian*”[tiab] OR “salvadorean*”[tiab] OR “salvadoran*”[tiab] OR “salvadorian*”[tiab] OR “nicaraguan*”[tiab] OR “south america*”[tiab] OR “bolivian*”[tiab] OR “chilean*”[tiab] OR “Chile”[Mesh] OR “colombian*”[tiab] OR “ecuadorian*”[tiab] OR “paraguay*”[tiab] OR “Paraguay”[Mesh] OR “venezuelan*”[tiab] OR “puerto rican*”[tiab] OR “puerto rico*”[tiab] OR “Puerto Rico”[Mesh] OR “spanish america*”[tiab] OR “boricua*”[tiab] OR “chicana*”[tiab] OR “chicano”[tiab] OR “Hispanic or Latino”[Mesh] OR “latinoamerican*”[tiab] OR “Mexican Americans”[Mesh] OR “spanish caribbean*”[tiab] OR “mexican american*”[tiab])
AND	
Cancer	(“Neoplasms”[Mesh] or neoplasm*[tw] or cancer*[tw] or tumor*[tw] or tumor*[tw] or malignan*[tw])
AND	
Genetic screening	((genetic[tw] or genetics[tw]) and (screening*[tw] or “Mass Screening”[Mesh])) OR “Genetic Testing”[Mesh] OR “Genetic Carrier Screening”[Mesh]
AND	
Publication date	(2005/1/1:2022/8/10[pdat])

**TABLE A2:** Search terms for Latin American and Caribbean Health Sciences Literature (LILACS).

Concept	Specific search terms
Cancer	cáncer o tumor
AND	
Genetics	genética/o
